# Exercise prior to assisted fertilization in overweight and obese women (FertilEX): study protocol for a randomized controlled trial

**DOI:** 10.1186/s13063-016-1398-x

**Published:** 2016-06-01

**Authors:** Kari Margrethe Lundgren, Liv Bente Romundstad, Vidar von Düring, Siv Mørkved, Sigrun Kjøtrød, Trine Moholdt

**Affiliations:** Department of Circulation and Medical Imaging, Faculty of Medicine, NTNU, Norwegian University of Science and Technology, Trondheim, Norway; Department of Fertility, St. Olavs Hospital, Trondheim University Hospital, Trondheim, Norway; Department of Public Health and General Practice, Faculty of Medicine, NTNU, Norwegian University of Science and Technology, Trondheim, Norway; Clinical Service, St. Olavs Hospital, Trondheim University Hospital, Trondheim, Norway; Department of Laboratory Medicine, Children’s and Women’s Health, Faculty of Medicine, NTNU, Norwegian University of Science and Technology, Trondheim, Norway

**Keywords:** Obesity, Infertility, High-intensity training, Polycystic ovary syndrome

## Abstract

**Background:**

Overweight and obese women show reduced conception rates compared to women of normal weight. Insulin resistance and increased amount of visceral fat may be important mechanisms for reduced fertility in these women. Exercise training, in particular with high intensity, has previously been found to improve insulin sensitivity in overweight subjects. This study will assess if regular high-intensity interval training will improve the pregnancy rate after assisted fertilization compared to usual care only in overweight and obese women. We hypothesize that the intervention will improve pregnancy rate and insulin sensitivity compared to the control group.

**Methods/design:**

The FertilEX study is a randomized, controlled trial in which 140 women with body mass index (BMI) >25 kg/m^2^ accepted for assisted fertilization will be randomized (1:1) to an intervention group or a control group. The intervention group will do high-intensity interval training three times per week for 10 weeks before assisted fertilization. The control group will receive standard care assisted fertilization only. The primary outcome measure is ongoing pregnancy 7–8 weeks after embryo transfer. Secondary outcome measures are insulin sensitivity, peak oxygen uptake, brachial flow-mediated endothelial function, levels of reproductive hormones, and body composition.

**Discussion:**

The results of this trial will provide knowledge about the effects of high-intensity exercise before assisted fertilization in subfertile overweight/obese women. If the intervention leads to beneficial effects on outcome measures, such programs should be considered as part of regular fertility care procedures for this population of women.

**Trial registration:**

ClinicalTrials.gov: NCT01933633. Registered on 28 August 2013.

**Electronic supplementary material:**

The online version of this article (doi:10.1186/s13063-016-1398-x) contains supplementary material, which is available to authorized users.

## Background

Obesity prevalence is increasing among women of reproductive age. This has evident consequences for public health, as obesity is associated with several chronic diseases and premature mortality [1, 2]. In addition, obesity is associated with an increased risk of subfertility [3]. Women who have a body mass index (BMI, in kg/m^2^) above 24 have a significantly elevated relative risk of subfertility compared to women with lower BMIs [4]. Although the exact mechanism through which fertility is affected by body mass and body fat is uncertain, adipose tissue has been shown to disturb the secretion and bioavailability of sex hormones and to affect egg quality, via effects of insulin, leptin, and adipokines [5, 6]. In addition to obesity alone, polycystic ovary syndrome (PCOS) is a common endocrine disorder affecting 6–20 % of women of reproductive age [7] and is currently recognized as the leading cause of anovulatory infertility.

The mechanisms underlying the intrinsic insulin resistance in PCOS remain unclear, but potentially they are associated with increased abdominal visceral fat [8]. In other insulin resistant populations, exercise training has consistently been shown to improve cardiovascular risk factors and reduce the risk of type 2 diabetes [9–11]. Furthermore, insulin sensitivity has been found to increase after a period of exercise training [10, 12]. Sim et al. [13] found significantly improved pregnancy rates after a 12-week weight loss program including exercise advice and diet intervention in obese women undergoing assisted fertilization. A recent observational study [14] also indicated that regular physical activity is associated with improved fertility in obese women going through fresh in vitro fertilization (IVF) or intracytoplasmic sperm injection (ICSI) cycles. Still, there is a considerable gap in the research literature regarding the effects of exercise training on fertility outcomes, and future research should focus on well-designed, adequately powered studies to address this gap [15]. In particular, research is needed on the isolated effects of exercise training, i.e., in the absence of dietary interventions. Also, there is a great need to understand the potential mechanisms through which exercise training can affect fertility.

In this article, we describe the design and methods of the FertilEX trial, a randomized controlled trial of exercise training prior to assisted fertilization in overweight and obese women.

The primary aim of the FertilEX trial is to test the hypothesis that overweight and obese women who exercise regularly prior to assisted fertilization will improve their pregnancy rate compared to women who only receive usual care assisted fertilization. The FertilEX trial will investigate possible mechanisms for increased fertility after exercising, such as insulin sensitivity, body composition and fat distribution, secretion and bioavailability of sex hormones, egg quality, cytokine levels, and blood markers of low-grade inflammation. Furthermore, we will assess the effects of exercise training on peak oxygen uptake, peripheral arterial endothelial function, and blood pressure.

## Methods

### Objectives

Our main hypothesis is that high-intensity interval training (HIT) before going into regular assisted fertilization with IVF or ICSI will improve the pregnancy rate in overweight and obese women. As a mechanistic explanation of the increased pregnancy rate, we expect that insulin sensitivity will improve in the women in the exercise group, and that the amount of reproductive and metabolic hormones will change towards normal values in these women.

### Participants and setting

The trial is conducted at St. Olavs Hospital, Trondheim University Hospital in collaboration with the Norwegian University of Science and Technology (NTNU), Trondheim, Norway. The trial includes women with a BMI >25 kg/m^2^ who are accepted for assisted fertilization at the St. Olavs Hospital. The trial was designed following the Standard Protocol Items: Recommendations for Interventional Trials (SPIRIT) 2013 statement (see Additional file 1).

Inclusion and exclusion criteria are listed in Table 1. To be included, the women must be willing to come to Trondheim for baseline assessments and post-testing after the intervention period. If allocated to the intervention group, the women must be willing to attend supervised training until they are familiarized with the training protocols and to continue to do HIT for 10 weeks.

**Table 1:** Inclusion and exclusion criteria

Inclusion criteria	Exclusion criteria
Age ≥18 years	Current high-intensity exercise training ≥2 times per week
Accepted for assisted fertilization	Current or previous Metformin® use (with wash-out period of >4 weeks)
BMI >25 kg/m^2^	Physical impairments limiting exercise training
Able to come for assessments at baseline and follow-up	Unable to follow verbal and written instructions in Norwegian or English
	Unwilling to suspend fertility treatment for 10 weeks

The recruitment started in November 2013 and will continue until the needed number of participants is included, anticipated until the end of 2018. Participating women in the training group get a free membership at a local gym during the intervention period, and women in the control group get a gift from the gym worth $85 US dollars.

### Randomization and allocation

**Fig. 1:**
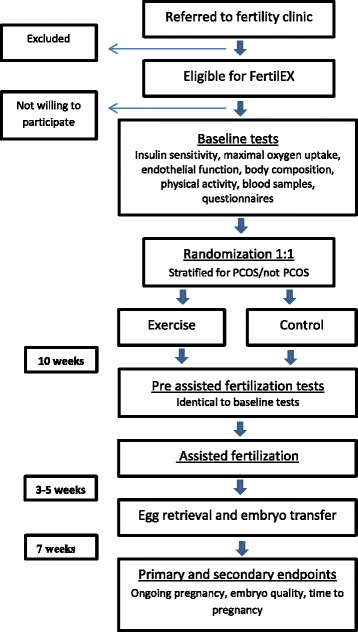
Participant flow through the FertilEX study

Participants are stratified for PCOS (yes/no) and randomly assigned to either the intervention or control group after initial assessments in a 1:1 manner, as outlined in the flow diagram (Fig. 1). Randomization is performed by a web-based randomization system developed and administered by the Unit of Applied Clinical Research, Institute of Cancer Research and Molecular Medicine, NTNU, Trondheim, Norway. The randomization is in blocks with varying block size.

### Intervention

**Fig. 2:**
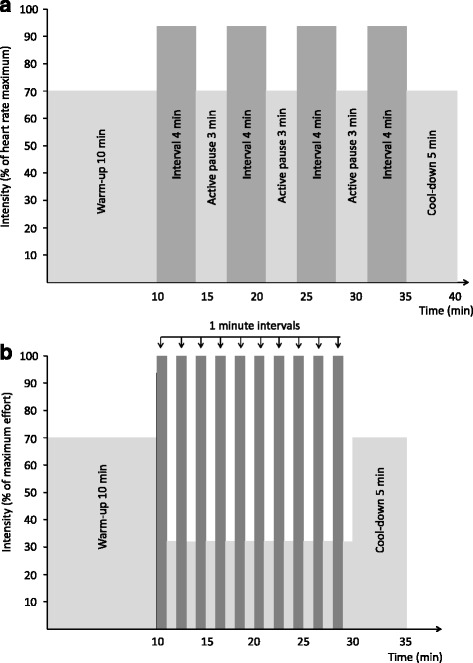
Training protocols. **a**) 4 × 4 min interval training. **b**) 10 × 1 min interval training

The exercise training program consists of HIT performed three times per week for 10 weeks. In two of the weekly sessions, the participants perform intervals with an intensity of 85–95 % of individual heart rate maximum for 4 minutes, repeated four times (Fig. 2a). In the third weekly exercise session, they perform intervals with maximum intensity that can be sustained for 1 minute, repeated 10 times (10 × 1) (Fig. 2b). All exercise training is preferably done as treadmill walking or running. In case of trouble with walking on a treadmill, other forms of exercise (bicycle, etc.) are used. This exercise regime has previously been found to improve insulin resistance by 17 % in women with PCOS [16]. The participants attend supervised exercise sessions until they are familiar with the training protocols. Thereafter, they continue exercising at the hospital or at a local gym. The participants wear heart rate monitors (Polar RCX3, POLAR, Oulu, Finland) at all training sessions to ensure that the exercise intensity is as prescribed. Heart rate data will be stored in a personal online training diary (http://www.polar.com) for use in analyses of compliance and exercise intensity. Exercise intensity is checked regularly throughout the intervention period by the study personnel. After the 10-week initial training period, all women follow standard care assisted fertilization, as described in the next paragraph. They are also encouraged to exercise as described above in the weeks of the fertility treatment until ovulation induction. Thus, the total exercise period is 13–15 weeks, depending on the fertility treatment protocol chosen by the treating physician. Women in the control group receive regular advice from the hospital staff about physical activity (usual care).

The fertility treatment includes an ovarian stimulation protocol, which is individualized for each patient according to standardized routines at the Fertility Unit, St. Olavs Hospital. We assume that the different protocols will be equally distributed in the interventional and control groups. The treatment includes either a short or a long stimulation protocol. The selection of protocol is done by the treating physician, who is blinded for group allocation. The treatment usually starts with a spontaneous onset of the period or by the use of progestins for women with long intervals between their menstrual bleedings. Prior to ovarian stimulation with gonadotropins, a urine hCG test to exclude pregnancy is taken by the patients at home. For the short ovarian stimulation protocol, one out of the three gonadotropins (Puregon®, Gonal-F®, or Menopur®) is administrated daily from cycle day 2 or 3. The antagonist (Orgalutran® 0.25 s.c.) is administered from the 5th treatment day. In the long gonadotropin releasing hormone (GnRH) agonist protocol, pituitary suppression is initiated with 800 μg nafarelin (Synarela® nasal spray) 5–7 days before the estimated start of the next menstrual bleeding and continued until the end of gonadotropin administration. Gonadotropin treatment starts after at least 14 days on GnRH agonist downregulation. Ovulation is induced by hCG injection (Ovitrelle 250 μg s.c.) when at least three follicles measure >17 mm. Ovum pick up is performed transvaginally 36 h later. The method used for fertilization is either IVF or ICSI. The day of embryo replacement is day 2, 3, or 5 after oocyte retrieval. Luteal phase support is given for 2 weeks. The patients take a urine hCG test 14 days after embryo replacement. For patients with a positive pregnancy test, a vaginal ultrasound examination is performed by 7 to 8 weeks of gestation.

### Study assessment visits

Participants come for research visits at baseline and after the intervention period (10 weeks) (Fig. 1). Clinical data collected by the hospital recordings during the treatment will be available for the study investigators.

### Outcome measures

The primary outcome measure is ongoing pregnancy, defined as the sonographic evidence of intrauterine gestational sac and fetal heart activity at 7 to 8 weeks of gestation. If not pregnant, the women will either do another IVF/ICSI cycle or are evaluated as not suitable for further treatment cycles, based on the treating physician’s decision. They will be considered as a negative for the primary outcome if not pregnant after the initial cycle.

Secondary outcome measures are insulin sensitivity, levels of reproduction-related hormones (luteinizing hormone (LH), follicle-stimulating hormone (FSH), prolactin, anti-Müllerian hormone (AMH), testosterone, free androgen index (FAI), sex hormone-binding globulin (SHBG)), peak oxygen uptake, brachial flow-mediated endothelial function, body weight, body composition, quality of life, physical activity, and diet. Also, the Fertility Unit systematically registers the number of eggs retrieved, the proportion of fertilized eggs and embryo quality, and pregnancy outcome data. These data will be available for the study investigators.

We measure insulin sensitivity by the gold standard, the hyperinsulinemic-euglycemic clamp, using a modification of the method originally described by De Fronzo et al. [17]. The subjects are placed in an adjustable bed in the supine position in a quiet and temperature stable room. Catheters are inserted in the right and left antecubital veins. The left catheter is used for blood sampling and the right for infusion of insulin and glucose. The left arm is placed in a heat package to enhance flow. Initial samples are collected to determine fasting glucose and insulin concentrations. After 30 min rest, human insulin (Actrapid®, Novo Nordisk, Bagsvaerd, Denmark), diluted in 500 mL NaCl 0.9 % to 300 mU/mL, is infused at a rate of 40 mU/m^2^/min, and maintained for the entire test. Plasma glucose concentration is measured every 5th minute (EKF Biosen C-line Sport, EKF diagnostic GmbH, Barleben, Germany) and kept at about 5.0 mmol/L for at least 30 min by adjusted infusion of 20 % glucose. Blood samples for assessing plasma insulin are taken at 30, 60, 90, and 120 min, as well as every 10th minute during the last 30 min of the test. The steady-state glucose infusion rate (GIR) is calculated for the last 30 min of the test, and the average GIR during this period will be used as the measure of insulin sensitivity. The homeostasis model assessment of insulin resistance (HOMA-IR) [18] will also be calculated.

Blood samples are obtained in the morning after a ≥10 h overnight fast and will be analyzed for concentrations of lipids, glucose, hemoglobin, glycated hemoglobin (HbA1c), albumin, high-sensitive C-reactive protein (CRP), and insulin C-peptide. The hormone assays include the evaluation of the above-mentioned reproduction-related hormones, as well as insulin, thyroid-stimulating hormone, leptin, and adiponectin. All analyses are conducted according to standard procedures at the Department of Medical Biochemistry, St. Olavs Hospital. Biological materials are stored at the Regional Biobank of Central Norway. Blood and urine samples are also collected and stored at the Regional Biobank of Central Norway for later analyses not yet planned. The Biobank is approved by the Data Inspectorate of Norway and by the Regional Committee for Medical Research Ethics. All information in the Biobank is handled according to the guidelines of the Data Inspectorate. Samples are stored with a unique identity number, corresponding to the participant’s identity number in the study, and with data on what the sample contains and when it was taken.

Peak oxygen uptake is measured in an incremental test to exhaustion by a direct ergospirometry system with a mixing chamber (Oxygen Pro, Erich Jaeger GmbH, Hoechberg, Germany) on a treadmill (Woodway USA Inc., Waukesha, WI, USA). A leveling off of oxygen uptake despite increased workload and a respiratory exchange ratio ≥1.05 are used as criteria for peak oxygen uptake. Measurements of peak oxygen uptake are performed at the core facility NeXt Move, Norwegian University of Science and Technology (NTNU).

Brachial flow-mediated endothelial function is measured by high-resolution vascular ultrasound (14 MHz Doppler probe, Vivid 7, GE Vingmed Ultrasound AS, Horten, Norway) according to current guidelines [19, 20]. The subjects are asked to refrain from strenuous physical activity for ≥48 h before the assessments, and measurements are obtained after a ≥10 h overnight fast. Measurements are conducted in a quiet and temperature stable room (22–24 °C) after 20 min of rest in a supine position. Baseline images are taken before occluding the forearm’s distal part by inflation of a pneumatic cuff (SC10, D.E. Hokanson, Inc., Bellevue, WA, USA) to 250 mmHg for 5 min. A longitudinal image of arterial diameter and pulse wave velocity signals is recorded continuously for 3 min after cuff release. An integrated electrocardiogram (ECG) is used to assess the diameter according to the cardiac cycle.

Body weight, fat mass, and fat-free mass are measured using bioelectrical impedance analysis (InBody 720, Biospace CO, Ltd., Seoul, Korea). Additionally, waist circumference is measured at the level of the umbilicus to further estimate the distribution of body fat.

Psychological well-being and quality of life are measured using standardized questionnaires, the Psychological General Well-Being Inventory [21] and the generic SF-36 quality of life questionnaire [22], respectively.

Physical activity is registered by questionnaires and by activity monitors (SenseWear, BodyMedia, Inc., Pittsburgh, PA, USA) at baseline and after 10 weeks. The subjects wear the activity monitor for five days: three weekdays and two weekend days. Diet is registered by an electronic standardized food frequency registration system [23] for two weekdays and one weekend day.

### Power calculation and statistical analyses

A clinical pregnancy rate of 0.30 during the first treatment cycle can be expected in this patient group. As the research hypothesis of this study is original, we have little previous data to support the power calculation. Based on a previous pilot study on giving advice about exercise and diet to improve pregnancy rates [24], we expect that the pregnancy rate in the intervention group will be increased to 0.55. With a statistical power of 0.80 and a significance level of 0.05, it is estimated that 61 patients are needed in each group to demonstrate the increased pregnancy rate. To allow for an expected 15 % dropout, we will include 140 participants in the study. We will analyze the data using the intention-to-treat principle (main analysis). Since dropout is one of the main concerns in studies of lifestyle modifications, we will also analyze the data with a per protocol analysis, excluding women in the intervention group who did not follow the prescribed intervention (defined as attending less than 80 % of the exercise sessions). As women in the control group might start exercising on their own, in the per protocol analyses we will exclude women in that group reporting to do high-intensity endurance training (making them breathe heavily) twice weekly or more during the intervention period. The proportion of women with ongoing pregnancy in the intervention and control groups will be compared using stratified (for PCOS/not PCOS) analysis of 2 × 2 tables. Missing data regarding pregnancy and fertilization outcome will be imputed with a negative result or zero; i.e., if no oocytes are collected during the assisted fertilization, the fertilization rate will be regarded as missing. The null hypothesis is that the proportion is equal in both groups, while the alternative hypothesis is that the proportion is larger in the intervention group. Secondary outcomes will be analyzed using analysis of covariance (ANCOVA), as suggested by Vickers and Altman [25]. We will use intervention group (exercise/control) as a fixed factor in the analyses and the baseline value of each outcome measure and PCOS status (PCOS/not PCOS) as covariates. When data on secondary outcomes are missing, we will use multiple imputation if we can assume that the data are missing at random. If we assume that the data are not missing at random, we will analyze only available data.

### Blinding

Baseline measurements, except for activity monitoring, are done before randomization. Assessments after the interventional period are done both blinded (insulin sensitivity, blood analyses, reproduction-related hormones, clinical data collected by the hospital during fertility treatment) and non-blinded (peak oxygen uptake, flow-mediated dilatation assessments). However, the analysis of the flow-mediated dilatation will be done blinded.

## Discussion

Lifestyle interventions, including regular exercise, healthful diet, and weight loss, are recommended to improve fertility in overweight and obese women [26]. At present, there are no evidenced-based guidelines on exercise training programs to improve fertility. We also need more knowledge about the underlying mechanisms for improvements in fertility after a period of exercise training. We have presented the rationale and design of a randomized controlled trial for investigating the effect of regular HIT in subfertile overweight/obese women. As insulin resistance and visceral fat deposition are thought to be central mechanisms contributing to decreased fertility in overweight/obese women [6, 27], we hypothesize that an intervention program designed to improve insulin sensitivity and reduce visceral fat will improve the success rate after assisted fertilization. We argue that this study will contribute important knowledge for further recommendations regarding lifestyle interventions in obesity-associated female subfertility.

To our knowledge, this is the first study of HIT as an approach to increase pregnancy rate among overweight and obese women referred to assisted fertilization. If successful, this study will bring new knowledge on preferred procedures to increase fertility rates in this population and offer guidance to clinicians treating these women.

### Trial status and change in study protocol after trial initiation

We are recruiting participants for the FertilEX study. As of March 2016, we have included 16 women in the study. We made a change to the study protocol on 16 February 2016, and from this time point on, the women allocated to the control group no longer wait for 10 weeks before the assisted fertilization treatment starts. This was done because we experienced that several women would not like to participate in the study as they felt that their treatment would be delayed. This study change therefore implies there will be no assessments after 10 weeks for the women in the control group. Also, as of February 2016, we started to include patients from a second fertility center, named Spiren Fertility Clinic, in Trondheim, Norway. This change was made to increase the recruitment to the study.

## Abbreviations

AMH, anti-Müllerian hormone; BMI, body mass index; ECG, electrocardiogram; FSH, follicle-stimulating hormone; GIR, glucose infusion rate; HIT, high-intensity interval training; HOMA-IR, homeostasis model assessment-estimated insulin resistance; ICSI, intracytoplasmic sperm injection; IVF, in vitro fertilization; LH, luteinizing hormone; PCOS, polycystic ovary syndrome; SHBG, sex hormone-binding globulin.

## Additional file

Additional file 1: SPIRIT 2013 Checklist: recommended items to address in a clinical trial protocol and related documents. (DOC 119 kb)
